# Indicanaemia

**Published:** 1918-05

**Authors:** 


**Indicanaemia.** Tcherkoff, quoted in Med. Rec., uses the following simple test: About 8-10 c.c. of blood serum is obtained in the usual way and treated with an equal quantity of 20% trichloracetic acid to precipitate protein and filtered. The clear liquid is tested like urine for indican, with strong HCl containing ½% of ferric chlorid, the color varying as for urine from a very pale up to a deep blue color. The rose or rose-violet color due to iodine may cause confusion so that, unless deliberately used as a rough test for iodine in the blood, all iodine medication should have been discontinued for some days before applying the test. As in the case of urine, quantitation by comparison of control color solutions may be employed. Normally, indican should be entirely absent and it is claimed that any appreciable coloration indicates an azotaemia (non-proteid) of 1.5% or more and is always due to renal insufficiency. (It may be questioned whether indican might not appear in the blood without renal insufficiency, in cases in which an excess of indol in the intestine is being rapidly excreted and showing as marked indicanuria.)

